# Incidence of peritoneal carcinomatosis after perioperative FLOT chemotherapy in gastric cancer. single-center analysis in Kazakhstan

**DOI:** 10.3389/fonc.2026.1684421

**Published:** 2026-02-10

**Authors:** Tomiris Sarina, Temirlan Kainazarov, Talgat Uskenbayev, Zhandos Burkitbayev, Sanzhar Shalekenov, Dinara Zharlyganova, Almira Manatova, Zhuldyz Kuanysh, Altay Kerimkulov

**Affiliations:** 1 Multidisciplinary Surgery Center, National Research Oncology Center, Astana, Kazakhstan; 2 Department of Specialized Medical Treatment, National Research Oncology Center, Astana, Kazakhstan; 3 Management Board, National Research Oncology Center, Astana, Kazakhstan; 4 Department of Scientific Management, National Research Oncology Center, Astana, Kazakhstan

**Keywords:** adjuvant chemotherapy, disease progression, FLOT, gastric cancer, neoadjuvant chemotherapy, peritoneal carcinomatosis

## Abstract

**Background:**

Peritoneal carcinomatosis (PC) significantly impacts prognosis in gastric cancer, limiting survival despite perioperative chemotherapy advancements. The FLOT regimen (5-fluorouracil, leucovorin, oxaliplatin, docetaxel) has become standard for resectable gastric cancer, yet real-world data on PC incidence and clinical impact of PC following FLOT-based treatment, particularly in Central Asia, are limited. This study aimed to evaluate the incidence and clinical implications of PC in a real-world cohort managed with a FLOT-based perioperative strategy in Kazakhstan.

**Methods:**

This retrospective cohort study included 74 with gastric cancer treated at the National Research Oncology Center, Kazakhstan (2020-2024). Patients were planned for perioperative FLOT-based systemic therapy followed by curative-intent surgery, with treatment adaptations reflecting routine clinical practice. Outcomes analyzed were overall survival (OS), relapse-free survival (RFS), and carcinomatosis-free survival (CFS), along with treatment response and patterns of recurrence.

**Results:**

The cohort was predominantly male (71.6%), with mean age 57.4 years. Most tumors (79.7%) were stage III at diagnosis. One- and two-year OS rates were 87.4% and 70.5%, respectively. RFS rates were 72.0% at one year, decreasing to 53.6% at two years. PC occurred in 50% of relapses, representing the most common recurrence pattern, with median post-progression survival of only 73.5 days. Carcinomatosis-free survival at one and two years was 87.8% and 77.9%, respectively. Prognostic analyses were exploratory due to limited event numbers. Clinical stage was not associated with carcinomatosis-free survival, whereas poorer tumor regression and signet-ring cell histology showed descriptively worse recurrence-free outcomes.

**Conclusions:**

Peritoneal carcinomatosis remains a significant challenge following perioperative FLOT chemotherapy in patients with gastric cancer in Kazakhstan, adversely influencing survival outcomes. These findings underscore the necessity for incorporating advanced diagnostic techniques, including peritoneal cytology and improved imaging methods, to enhance early detection and accurate staging. Further prospective studies are warranted to validate these results and develop tailored therapeutic strategies specific to regional healthcare settings.

## 1 Introduction

Globally, gastric cancer ranked fifth in incidence with 968,784 new cases (4.9%) and was responsible for 660,175 deaths (6.8%), making it the fifth leading cause of cancer-related mortality (1). In Kazakhstan in 2024, gastric cancer ranked fourth in incidence (2,843 new cases; 8.5%) and second in mortality (1428 deaths; 11.2%), highlighting its significant contribution to the national cancer burden (2). The presented data emphasize the high significance of gastric cancer for the healthcare system of Kazakhstan, especially in the context of regional epidemiological vulnerability and limited coverage of screening programs (3).

Despite advances in diagnostic tools and multimodal treatment, the prognosis for patients with advanced or recurrent disease remains poor - particularly in cases with peritoneal dissemination, which is often indicative of systemic treatment failure (4).

Peritoneal carcinomatosis (PC) is a common and devastating pattern of recurrence in gastric cancer, associated with rapid disease progression, limited therapeutic options, and a median survival of only 2 to 9 months (5–7). Studies have shown that PC is the most frequent site of recurrence following curative surgery in stage II and III gastric cancer, with rates of 33.3% and 38.7%, respectively, while being less common in stage I disease (14%) (8). Once established, it is rarely amenable to curative treatment and significantly compromises patient outcomes.

The perioperative FLOT regimen-comprising 5-fluorouracil, leucovorin, oxaliplatin, and docetaxel has become the standard of care for patients with resectable gastric or gastroesophageal junction adenocarcinoma, offering improved pathological response rates and overall survival compared to previous regimens such as ECF/ECX (9–11). In a large real-world cohort study, FLOT regimen showed the 2-year overall survival (OS) and disease-free survival (DFS) rates of 71.0% and 60.0% while 47.0% and 42.0% for the 5-FU/platinum group (12). However, in clinical practice, when standard regimens are not applicable, it is permissible to individually consider alternative regimens, such as DOC or EOF, whose efficacy and safety have also been confirmed in a number of studies (13, 14). Although these regimens are not part of the current international guidelines, they may be considered on a case-by-case basis, particularly in settings with limited resources or specific contraindications. The decision to use such alternatives should be based on the patient’s general condition, comorbidities, and the individual risk of treatment-related toxicity. However, while FLOT has been widely adopted in clinical practice, data regarding its impact on recurrence patterns, particularly the incidence of peritoneal carcinomatosis, remain limited.

While several studies have examined gastric cancer mortality trends, multimodal treatment outcomes, and other clinical aspects, there is a lack of real-world evidence evaluating the incidence of PC following FLOT chemotherapy – particularly in Central Asia (15–17), where gastric cancer remains a major public health concern. Patients from this region are often underrepresented in international studies, limiting the applicability of existing findings to local clinical practice.

To address this gap, we conducted a single-center analysis at a leading cancer institute in Kazakhstan, evaluating treatment outcomes in patients with resectable gastric cancer who received perioperative FLOT chemotherapy. Our study includes data on treatment response, progression-free survival (PFS), overall survival (OS), and carcinomatosis-free survival (CFS), with a specific focus on the incidence and clinical implications of peritoneal carcinomatosis following FLOT.

The aim of this study is to determine the incidence and prognostic significance of peritoneal carcinomatosis after perioperative FLOT chemotherapy and to evaluate its association with survival outcomes in a real-world Kazakhstani cohort, thereby contributing to the global understanding of recurrence and survival dynamics in gastric cancer.

## 2 Methods

### 2.1 Study design and population

This retrospective real-world cohort study included all consecutive patients with histologically confirmed gastric adenocarcinoma who were treated at the National Research Oncology Center in Astana, Kazakhstan, between January 2020 and January 2024. A total of 74 patients who underwent gastrectomy with curative intent and planned for a FLOT-based perioperative treatment strategy, with real-world deviations were identified from institutional medical records. Clinical, pathological, treatment, and follow-up data were extracted retrospectively and cross-verified independently by two investigators.

Eligibility for surgery and perioperative chemotherapy was determined by a multidisciplinary tumor board and was based on ECOG performance status (≤1), imaging findings (thoracoabdominal CT), and laboratory parameters (including liver and renal function tests). All patients underwent pre-treatment staging with upper endoscopy and histological confirmation of gastric adenocarcinoma.

Therapeutic choices were made according to national protocols and aligned with NCCN and ESMO guidelines. Patients were stratified based on clinical staging, comorbidity profiles, and institutional eligibility criteria for specific chemotherapy regimens. The study design was retrospective and patients were not randomized; however, treatment allocation was based on standardized criteria.

### 2.2 Statistical analysis

All statistical analyses were performed using R software (version 2024.12.1 + 563). Descriptive statistics were used to summarize baseline characteristics. Continuous variables were presented as means with standard deviations (SD), and categorical variables as frequencies and percentages.

OS, relapse-free survival (RFS), and post-progression survival (PPS) were estimated using the Kaplan-Meier method. Survival outcomes were reported at 1- and 2-year intervals with corresponding 95% confidence intervals (CIs). Time-to-event outcomes were recorded in days and converted to months when appropriate. Patients without the event of interest at the time of last follow-up were censored.

Associations with carcinomatosis-free survival (CFS) were explored using univariable Cox proportional hazards analyses due to the limited number of events. Hazard ratios (HRs) with 95% confidence intervals (CIs) were reported, with statistical significance defined as a two-sided p-value < 0.05.

### 2.3 Follow-up and monitoring

Patients were monitored after FLOT chemotherapy and radical surgery, then referred to a local outpatient clinic for observation after 3, 6, 9 months and then once a year. Follow-up evaluations included clinical examinations, contrast-enhanced CT scans or PET/CT imaging to detect disease recurrence or progression. The primary outcome was OS, defined as the time from diagnosis to death from any cause. Secondary outcomes included RFS, defined as the time from surgery to the first documented recurrence, and PPS, calculated from the date of disease progression to death. Disease recurrence was stratified by site (peritoneum, liver, lungs, or other). Tumor regression was assessed histologically using the Ryan Tumor Regression Grading (TRG) system. Clinical and pathological TNM stages were compared to assess staging accuracy. Patients lost to follow-up were censored at the time of their last documented clinical visit.

## 3 Results

### 3.1 Patient demographics and baseline clinical characteristics

A total seventy-four patients were included in the analysis after radical treatment (**Table 1**). The cohort was predominantly male (71.6%, n = 53), with the mean age was 57.4 years ± 8.8 years and a mean BMI of 26.0 ± 4.6 kg m-2. Baseline hemoglobin averaged 125 ± 17 g L-1 and total serum protein 68.4 ± 7.0 g L-1. Renal indices were within normal limits (urea 5.0 ± 1.5 mmol L-1; creatinine 72 ± 16 µmol L-1). The median hospital stay was 10 days (IQR 6-16). Comorbidity was limited: diabetes in 6.8% and “other” conditions in 27.0%. Anemia was present in 44.6%.

**Table 1:** Patient Demographics and Baseline Clinical Characteristics (n = 74).

Variable	n (%) or mean ± SD
Demographics	
Age (years) (mean ± SD)	57.4 ± 8.8
BMI (kg/m²) (mean ± SD)	26.0 ± 4.6
Sex (%)	
Male	53 (71.6%)
Female	21 (28.4%)
Tumor Characteristics	
Tumor Localization	
Upper third	35 (47.3%)
Middle third	25 (33.8%)
Lower third	14 (18.9%)
Clinical T stage (cT)	
T2	3 (4.1%)
T3	59 (79.7%)
T4	12 (16.2%)
Clinical N stage (cN)	
N0	11 (14.9%)
N+	58 (78.4%)
Nx	5 (6.8%)
Clinical Stage (AJCC 8th ed.)	
Stage II	15 (20.3%)
Stage III	59 (79.7%)

Tumors most frequently arose in the upper third of the stomach (47.3%), 33.8%(n=25) in the middle third, and 18.9% (n=14) in the lower third. The clinical staging at diagnosis showed that most patients were at advanced T-stages, with 79.7% (n=59) at cT3, 16.2% (n=12) at cT4, and only 4.1% (n=3) at cT2. Lymph node involvement was present in the majority, with 78.4% (n=58) being N+, while 14.9% (n=11) were N0, and 6.8% (n=5) had unknown nodal status (Nx). Based on clinical staging, 79.7% (n=59) of patients were at Stage III, and 20.3% (n=15) at Stage II.

Among our patients, histological grading ranged from G1 to G4, as determined by endoscopic biopsy and subsequently confirmed by postoperative pathological examination or immunohistochemical examination, in the case of G4. Emerging evidence suggests that microsatellite instability, HER2 overexpression, and claudin 18–2 expression represent clinically relevant biomarkers that could guide the use of targeted and immunotherapeutic strategies in advanced gastric cancer (18). However, HER2 status, microsatellite instability, and claudin 18–2 expression were not assessed in this retrospective study because these molecular markers were not routinely tested at the time of diagnosis in our center.

### 3.2 Treatment exposure

Neoadjuvant chemotherapy (N-CTx) was completed as planned in 86.5%, with 81.1% receiving the full four cycles of FLOT. The predominant surgical procedure was total gastrectomy (74.3%), followed by subtotal gastrectomy (14.9%) and Ivor Lewis Esophagectomy (4.0%). Five patients (6.8%) underwent palliative surgical procedures or biopsy due to disease progression detected intraoperatively. Post-operative R0 resection margins were achieved in 82.4%. Adjuvant chemotherapy was administered to 79.7% - most commonly FLOT (47.3%) (**Table 2**).

**Table 2:** Treatment Details and Pathological Findings (N = 74).

Variable	n (%)
Neoadjuvant Chemotherapy	
Number of FLOT cycles	
4 cycles	60 (81.1%)
<4 cycles	9 (12.2%)
Other / Unknown	5 (6.8%)
Neoadjuvant CTx completion	
Complete	64 (86.5%)
Incomplete	9 (12.2%)
Adjuvant Chemotherapy	
Received Adjuvant CTx	
Yes	59 (79.7%)
No	15 (20.3%)
Adjuvant CTx Regimen	
FLOT	35 (47.3%)
Other regiments	24 (32.4%)
Surgical Details	
Type of Surgery	
Total gastrectomy (TG)	55 (74.3%)
Subtotal gastrectomy (STG)	11 (14.9%)
Ivor Lewis Esophagectomy	3 (4.0%)
Palliative surgery or biopsy	5 (6.8%)
Pathological Findings (ypTNM)	
Resection margin (R status)	
R0	61 (82.4%)
R1	8 (10.8%)
NA	5 (6.8)
ypT (%)	
T0	3 (4.1)
Т1	6 (8.1)
T2	9 (12.2)
T3	33 (44.6)
T4	16 (21.6)
NA	7 (9.5)
ypN (%)	
N0	25 (33.8)
N1	14 (18.9)
N2	14 (18.9)
N3	14 (18.9)
NA	7 (9.5)
Histopathological Tumor Regression *	
Grade 1	22 (29.7%)
Grade 2	10 (13.5%)
Grade 3	22 (29.7%)
Grade 4	3 (4.1%)
Grade 5	3 (4.1%)
no regression	3 (4.1%)
no data	6 (8.1%)
NA	5 (6.8%)

*Ryan Tumor Regression Grading (TRG) system.

Adjuvant chemotherapy was administered to 79.7% of patients, most commonly FLOT (47.3%), while 32.4% received alternative regimens, including XELOX, FOLFOX and ECF (**Table 2**).

Deviations from the planned perioperative FLOT strategy reflected real-world clinical practice. Five patients demonstrated disease progression intraoperatively and therefore did not receive systemic chemotherapy. Ten patients did not receive adjuvant chemotherapy due to patient refusal (n = 2), absence of indication after downstaging (n = 1), postoperative mortality (n = 2), severe toxicity or complications following neoadjuvant chemotherapy (n = 3), or completion of extended perioperative neoadjuvant treatment (eight cycles) without planned adjuvant therapy (n = 2).

### 3.3 Survival outcomes

**Figure 1:**
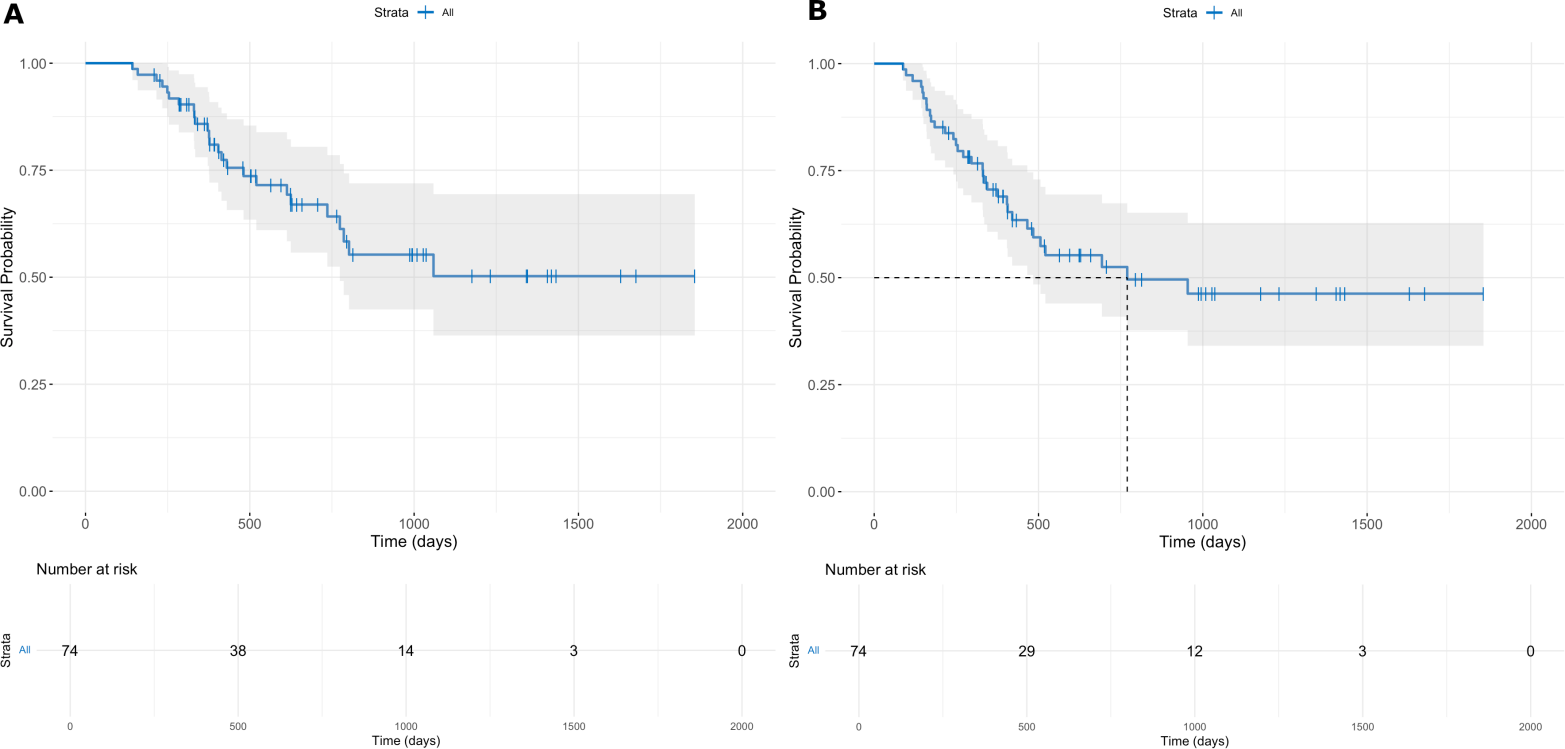
One- and two-year OS **(A)** and RFS **(B)**. No median survival line shown for OS as 50% survival was not reached.

Median follow-up was 22 months (range 2-48). One- and two-year OS were 87.4% (95% CI: 79.9-95.4) and 70.5% (95% CI: 59.7-83.4), respectively. RFS rates were 72.0% (95% CI: 62.3-83.3) and 53.6% (95% CI: 41.9-68.5). Median OS and RFS were not reached by the analysis cut-off. The survival curves are presented in **Figure 1**.

CFS was assessed, showing that 87.8% of patients remained free of peritoneal carcinomatosis at 1-year post-diagnosis (95% CI: 79.1-97.6), declining to 77.9% (95% CI: 65.4-92.7) at 2 years.

#### 3.3.1 Subgroup survival analysis by clinical stages

**Figure 2:**
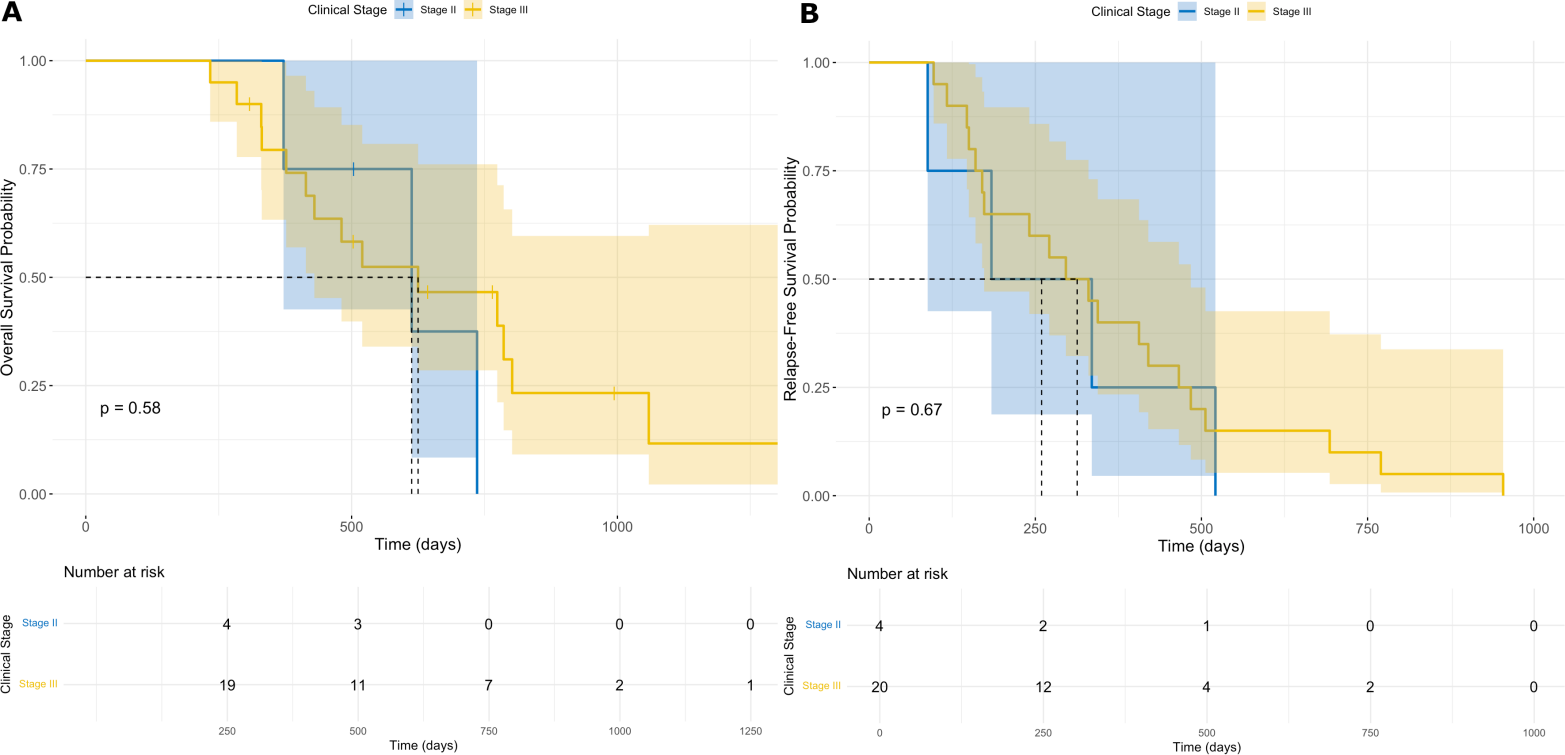
One- and two-year OS **(A)** and RFS **(B)** by clinical stages.

Stage II patients achieved a 1-year OS of 100% but fell to 37.5% by 2 years; corresponding RFS rates were 25.0% and 0%. Stage III patients showed 1-/2-year OS of 79.4%/46.6% and RFS of 40.0%/10.0% (**Figure 2**). The results obtained may, in part, be attributed to the imbalance in patient distribution across disease stages, with only 15 patients in the stage II cohort compared to 59 in the stage III group. Additionally, the potential for staging inaccuracy due to preoperative CT-based assessment should not be overlooked, as this imaging modality may contribute to a certain degree of misclassification (19). Moreover, tumor biology - specifically the degree of aggressiveness - was not captured in the presented table or included in this particular analysis, which may also influence the observed outcomes.

### 3.4 Patterns of disease progression and post-progression survival

Patterns of disease progression were analyzed at the patient level. Recurrences were first categorized as single-site or multi-site events. For survival analyses after progression, each patient was assigned a dominant recurrence site based on clinical documentation; cases involving peritoneal carcinomatosis were classified as PC-dominant. Dominant site was defined as the clinically leading site of recurrence as documented in radiology reports and multidisciplinary discussion.

Among the 24 patients who experienced disease progression, peritoneal carcinomatosis was the dominant recurrence pattern in 50%, followed by liver metastases in 25%, other non-visceral or locoregional progression in 16.7%, and lung metastases in 8.3% (**Table 3**).

**Table 3:** Survival after progression by dominant recurrence pattern.

Dominant recurrence site	n	%	Median PPS (days)	Mean PPS (days)
Peritoneal Carcinomatosis (± other sites)	12	50.0%	73.5	84.4
Liver Metastasis (without PC or lung)	6	25.0%	505	555
Lung Metastasis (without PC or liver)	2	8.3%	164	164
Other Progressions	4	16.7%	315.5	308.3

*PPS, post-progression survival; PC, peritoneal carcinomatosis.

Post-progression survival differed substantially according to the dominant site of recurrence. Median post-progression survival was 73.5 days for peritoneal carcinomatosis, 164 days for lung metastases, 316 days for other progression patterns, and 505 days for liver metastases. These findings highlight the particularly poor prognosis associated with peritoneal recurrence and underscore the clinical relevance of progression patterns in shaping post-progression outcomes.

### 3.5 Prognostic factors

Given the limited number of PC events, multivariable regression modeling was not performed. Associations with CFS were explored using univariable analyses only and should be interpreted as descriptive and hypothesis-generating. In univariable Cox analysis, clinical stage (Stage III vs Stage II) was not significantly associated with CFS (HR 2.34; 95% CI 0.30–18.21; p = 0.416), with wide confidence intervals reflecting limited statistical precision.

Formal Cox regression modeling of histopathological tumor regression grade (TRG) was not feasible due to sparse events across multiple TRG categories and evidence of quasi-complete separation. Therefore, the prognostic impact of TRG was evaluated descriptively. Patients with poorer regression grades and signet-ring cell histology demonstrated inferior recurrence-free survival, while overall survival differences were less pronounced; however, these observations are limited by small subgroup sizes and should be considered exploratory.

## 4 Discussion

This single-center retrospective study provides real-world data on the incidence and clinical impact of PC following perioperative FLOT chemotherapy in gastric cancer patients in Kazakhstan. Our cohort represents a real-world population treated with a FLOT-based perioperative strategy rather than a strictly protocolized perioperative FLOT trial cohort. To our knowledge, no prior studies have specifically examined peritoneal recurrence rates after perioperative chemotherapy in Central Asia. While a retrospective analysis of Kazakhstani patients reported a 19% progression rate and 1- and 3-year RFS of 76.8% and 39%, respectively (20), data regarding PC incidence remain absent. Our findings, therefore, help bridge this region-specific gap.

The observed PC incidence of 50% in our cohort aligns with international studies reporting PC recurrence rates between 44.1% and 56% after perioperative chemotherapy (21, 22), underscoring the persistent challenge of peritoneal dissemination despite systemic control achieved with modern regimens such as FLOT. In terms of survival, our 2-year OS rate of 70.5% and RFS of 53.6% are consistent with outcomes reported in pivotal studies. The FLOT4 trial demonstrated a 2-year OS of approximately 71.0% and DFS of 60.0% (9), while Möhring et al. reported a median OS of 57.8 months in real-world settings (12). However, a notable disparity between OS and RFS was observed, particularly in stage II patients, where RFS dropped from 25.0% at one year to 0% at two years, suggesting either aggressive disease biology or limitations in staging accuracy.

This study is among the first in Central Asia to report CFS as a distinct outcome metric. We found a 1-year CFS rate of 87.8% (95% CI: 79.1-97.6%) and a 2-year rate of 77.9% (95% CI: 65.4-92.7%). Clinically, this is significant: PC was the most frequent and lethal site of progression, with a median post-progression survival of only 73.5 days - consistent with previous literature identifying PC as the earliest and most aggressive form of recurrence (23, 24).

Histopathological tumor regression emerged as a potential protective factor against PC, though without reaching statistical significance. This aligns with the hypothesis that effective systemic chemotherapy, such as FLOT may reduce microscopic peritoneal dissemination. While systemic chemotherapy has limited efficacy against established peritoneal metastasis due to poor peritoneal-plasma penetration (8), its role in targeting subclinical disease is supported by improved RFS in randomized trials and reflected in international guidelines (9, 25). Despite FLOT’s systemic efficacy, the development of PC remains a common and devastating form of recurrence. Mechanistically, peritoneal dissemination occurs via transcoelomic spread, mesothelial adhesion, and stromal invasion (26). Additionally, chemotherapy-resistant clones, particularly in diffuse or signet-ring cell carcinomas, may evade treatment due to their mesenchymal phenotype and strong peritoneal tropism, likely contributing to the high PC rates observed in our cohort.

In advanced or peritoneal disease, alternative systemic regimens such as docetaxel-based combinations demonstrate comparable efficacy with differing toxicity profiles, supporting individualized treatment selection (13). Given the intensity and potential toxicity of perioperative FLOT chemotherapy, particularly in patients with advanced or borderline performance status, nutritional assessment and support should be considered as part of a multidisciplinary approach. Emerging evidence suggests that nutritional status may significantly influence treatment tolerance and oncologic outcomes in gastric cancer patients, highlighting the value of early involvement of a clinical nutritionist (27). While epirubicin-containing regimens (e.g., ECF, EOF) were previously standard (28), they are no longer recommended in current NCCN or ESMO guidelines due to limited survival benefit and increased toxicity. In selected patients with limited peritoneal carcinomatosis, cytoreductive surgery combined with HIPEC may be considered (29); however, randomized evidence such as the GASTRIPEC-I trial has failed to show a significant overall survival benefit from the addition of HIPEC (30).

Notably, clinical staging based on CT imaging failed to predict PC effectively. This is consistent with studies highlighting the limited sensitivity of conventional imaging for detecting micrometastatic peritoneal disease (31). In our retrospective study, resources such as peritoneal washing cytology and diagnostic laparoscopy were not routinely available. However, these methods are now standard practice in many centers and play a crucial role in improving the detection of occult peritoneal involvement. The lack of such diagnostic tools at the time likely reduced staging accuracy, leading to underestimation of the peritoneal carcinomatosis burden and impacting both staging and treatment decisions (32). Further complicating staging accuracy was the limited availability of PET/CT and reliance on abdominal ultrasonography, which has significantly lower sensitivity and specificity than advanced imaging modalities (33). These infrastructural constraints may have led to underestimation of the true PC burden and impacted both reported incidence and survival outcomes. Subgroup analysis revealed a nearly threefold increased risk of PC in patients with signet-ring cell histology, corroborating previous studies that emphasize the aggressive nature and peritoneal affinity of this subtype. The FLOT4 trial also reported limited benefit of FLOT in signet-ring cell carcinoma, suggesting the need for histology-tailored treatment strategies (9).

Limitations of this study include its retrospective design, which is inherently subject to selection bias, incomplete data, and lack of standardized follow-up. In Kazakhstan, the absence of a centralized cancer registry and national follow-up protocols significantly hampers long-term data collection, a contrast to surveillance systems in high-income countries (34, 35). These systemic gaps not only impact patient care but also limit the precision of real-world oncology research in the region. Moreover, the lack of peritoneal washing cytology and laparoscopy likely led to underdiagnosis of occult peritoneal involvement, influencing both staging and treatment decisions. Although the study included consecutive patients receiving a standardized FLOT protocol, which partially mitigates selection bias, the results must still be interpreted with caution.

Despite these limitations, our study offers important real-world insights into gastric cancer management in Central Asia. The findings highlight a critical need for improved diagnostic infrastructure, standardized staging protocols, and longitudinal patient monitoring to optimize outcomes and inform future regional cancer control strategies.

## 5 Conclusion

Peritoneal carcinomatosis remains a major contributor to poor outcomes in gastric cancer patients receiving perioperative FLOT chemotherapy. Our findings, derived from a Central Asian population, emphasize the need for enhanced diagnostic staging through peritoneal cytology and advanced imaging. While these results provide important real-world insights, they should be interpreted with caution given the retrospective design. Future prospective studies should prioritize the integration of these tools to improve early detection, refine risk stratification, and guide therapeutic decisions more effectively. Moreover, further multicenter prospective studies are warranted to validate these observations and support potential inclusion in future clinical guidelines.

## Data Availability

The raw data supporting the conclusions of this article will be made available by the authors, without undue reservation.
